# Variant calling from scRNA-seq data allows the assessment of cellular identity in patient-derived cell lines

**DOI:** 10.1038/s41467-022-30230-w

**Published:** 2022-05-12

**Authors:** Daniele Ramazzotti, Fabrizio Angaroni, Davide Maspero, Gianluca Ascolani, Isabella Castiglioni, Rocco Piazza, Marco Antoniotti, Alex Graudenzi

**Affiliations:** 1 Dept. of Medicine and Surgery, Univ. of Milan-Bicocca, Monza, Italy; 2 Dept. of Informatics, Systems and Communication, Univ. of Milan-Bicocca, Milan, Italy; 3 Inst. of Molecular Bioimaging and Physiology, National Research Council (IBFM-CNR), Segrate, Milan Italy; 4 Dept. of Physics “Giuseppe Occhialini”, Univ. of Milan-Bicocca, Milan, Italy; 5 Bicocca Bioinformatics, Biostatistics and Bioimaging Centre – B4, Milan, Italy

**Keywords:** Cancer genomics, Data integration

**arising from** Sharma et al. *Nature Communications* 10.1038/s41467-018-07261-3 (2018)

Single-cell sequencing experiments enable the investigation of cell-to-cell heterogeneity at unprecedented resolution^1^, and this is especially relevant in the study of cancer evolution^2^. In ref. ^3^, the authors employed longitudinal single-cell transcriptomic data from patient-derived primary and metastatic Oral Squamous Cell Carcinomas (OSCC) cell lines (from a previous panel^4^), to investigate possible divergent modes of chemo-resistance in tumor subpopulations. We integrated the analyses by performing variant calling from single-cell RNA sequencing (scRNA-seq) data via GATK Best Practices^5^, and discovered a high number of Single-Nucleotide Variants (SNVs) representative of the identity of a specific patient in the cell line derived from a second patient, and vice versa. These findings suggest the existence of a sample swap, thus jeopardizing some key translational conclusions of the article, and prove the efficacy of a joint analysis of the genotypic and transcriptomic identity of single cells.

Even though scRNA-seq data are typically employed to characterize single-cell gene expression profiles^6^, recent studies proved that data generated with full-length protocols (e.g., Smart-Seq/Smart-Seq2^7^) can be effectively used for variant calling^8^. Despite known pitfalls, such as the impossibility of calling genomic variants from non-transcribed regions and the high rates of noise and dropouts^9^, this provides a highly-available and cost-effective alternative to DNA sequencing^10^. The mutational profiles so obtained can be used to determine the identity of single cells, and this is useful to characterize the clonal evolution of tumors^11^ and assess the impact of therapies, when longitudinal experiments are available^12^. Furthermore, this allows a natural mapping between the genotype and the gene expression profile of single cells^13^. This aspect has significant translational relevance, given the shortage of accurate and affordable technologies for concurrent DNA and RNA sequencing of the same cells, despite the introduction of new protocols^14,15^.

We integrated the analyses presented in^3^ and selected the scRNA-seq datasets of two cell lines derived from distinct OSCC patients (HN120 and HN137) which include different data points, marked with the suffixes: -P (primary line), -M (metastatic line), -CR (after cisplatin treatment), -CRDH (after drug-holiday). Since, for the HN137P cell line, single- and paired-end library layouts are provided, and HN137MCRDH is not present, we have a total of 12 datasets (GEO accession code GSE117872; refer to^3^ for details on the experimental setup). In detail, we selected single cells labeled as “good data” and performed variant calling with the procedure employed in^12^ and described in the Supplementary Information (SI).

4, 924, 559 unique variants were detected on a total of 1, 116 single cells included in all datasets. Quality control filters were applied to ensure high confidence to the calls and reduce the number of false alleles and miscalls. In particular, we removed: (i) indels and other structural variants—to limit the impact of sequencing and alignment artifacts, (ii) variants mapped on mitochondrial genes, (iii) variants on positions with coverage < 5 reads in > 50% of the cells in each time point—to focus the analysis on well-covered positions, (iv) variants detected in less than 20% of both HN120P and HN137P *(single-end)* cells—to focus on recurrent variants, (v) variants detected (≥3 reads supporting the alternative allele) in both HN120P and HN137P *(single-end)*—to define a list of variants clearly characterizing the identity of the two primary cell lines. We finally selected the variants observed in at least 1 cell (≥3 reads supporting the alternative allele, ≥5 coverage) of HN120P and in exactly 0 cells of HN137P *(single-end)*, and the variants observed in at least 1 cell (≥3 reads supporting the alternative allele, ≥5 coverage) of a HN137P *(single-end)* and in exactly 0 cells of HN120P.

As a result, we identified 67 SNVs representative of HN120P cell identity. Such variants are observed at high frequency in HN120P and in HN137P *(paired-end)*, HN137PCR, HN137PCRDH, HN137M, HN137MCR, whereas are not observed (<1% of the cells) in HN120PCR, HN120PCRDH, HN120M, HN120MCR, HN120MCRDH and HN137P *(single-end)*. In Fig. 1A, we display the mutational profiles of all single cells in all datasets (coverage information is provided in Supplementary Data 1).

**Fig. 1:**
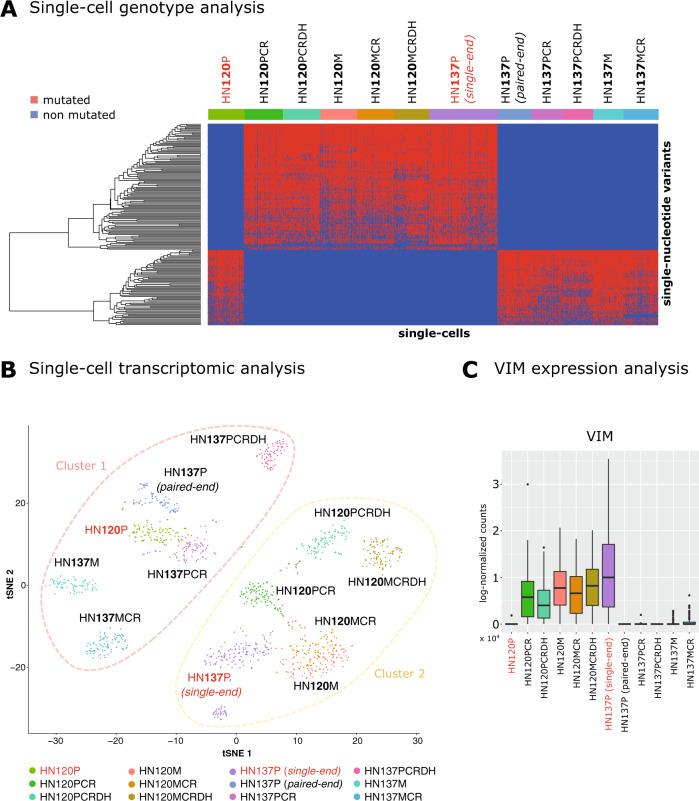
Analysis of single-cell mutational and gene expression profiles of patient-derived OSCC cell lines from scRNA-seq data. **A** The heatmap including the mutational profiles of all single cells of the HN120 and HN137 datasets is displayed (-P: primary line, -M: metastatic line, -CR: after cisplatin treatment, -CRDH: after drug-holiday). Red entries mark cells displaying a given SNV. For the ID of single cells and SNVs please refer to Supplementary Data 1 and 2. **B** The t-SNE plot generated from the gene expression profiles of all single cells for all datasets is shown (see the SI for additional details). **C** The distribution of the expression level of *VIM* on all single cells is shown with boxplots for all datasets.

Analogously, we identified 112 SNVs that are strongly informative for HN137P *(single-end)* identity (see Fig. 1A). Such variants are observed at high frequency in HN137P *(single-end)* and in HN120PCR, HN120PCRDH, HN120M, HN120MCR, HN120MCRDH, whereas are not observed (<1% of the cells) in HN137P *(paired-end)*, HN137PCR, HN137PCRDH, HN137M, HN137MCR, and HN120P. The attributes of the SNVs are reported in Supplementary Data 2.

From the analysis, it is evident that the genotypic identity of HN120P cell line is inconsistent with that of the other HN120 datasets and with that of HN137P *(single-end)*, whereas it is consistent with that of the remaining HN137 datasets. Conversely, the genotypic identity of HN137P *(single-end)* cell line is inconsistent with that of the other HN137 datasets and with that of HN120P, while being consistent with that of all the other HN120 datasets. This consideration holds whether such SNVs are either germline or somatic, as genotypes are unquestionable footprints of cell identity (notice also that 177 over 179 variants have a rsID). These surprising results can be hardly explained by cancer-related selection phenomena, random effects, or sampling limitations. Instead, this suggests the likely presence of a methodological issue involving a label swap of samples HN120P and HN137P *(single-end)*.

This hypothesis is further supported by the single-cell transcriptomic analysis performed via Seurat^16^ (see the SI). In Fig. 1B, one can find the t-SNE plot computed on the 1000 most variable genes. Consistently with the genotype analysis, the transcriptomic analysis highlights the presence of two distinct clusters, the first one including HN120P cells and all cells from HN137 datasets, excluded HN137P *(single-end)*, the second one including HN137P *(single-end)* cells and all cells from HN120 datasets, excluded HN120P.

Unfortunately, we believe that this methodological error may have led to erroneous conclusions in refs. ^3,17,18^. In^3^, for instance, the authors state that HN137 cell line is comprised of a mix of epithelial (*ECAD*+) and mesenchymal (*VIM*+) cells, whereas the HN120 cell line would include phenotypically homogeneous population of *ECAD*+ cells. However, by looking at the expression level of *VIM* (Fig. 1C), one can notice that this gene is up-regulated in HN137P *(single-end)* and in all HN120 datasets, excluded HN120P, whereas is down-regulated (median = 0) in HN120P and in all HN137 datasets, excluded HN137(*single-end*).

Furthermore, in^3^ the authors state that, in presence of cisplatin treatment, the heterogeneous HN137P cells demonstrate a progressive enrichment of *ECAD*, and the gradual depletion of *VIM*+ cells, until the latter gets extinct. Conversely, from the supposedly homogeneous *ECAD*+ population of HN120P cells, the authors report the de novo emergence of *VIM*+ cells after two weeks of treatment. To explain this unexpected phenomenon, the authors invoke the presence of a covert epigenetic mechanism that emerges under drug-induced selective pressure. Instead, we believe that this result might be easily explained by a label swap of HN120P and HN137P *(single-end)*, as confirmed by the analyses presented above.

Overall, our results prove that scRNA-seq data can be effectively exploited to perform an integrated analysis of the genotypic and transcriptomic identity of single cells, providing a powerful tool to decipher complex phenomena such as cancer evolution and drug resistance.

## Reporting summary

Further information on research design is available in the Nature Research Reporting Summary linked to this article.

## Supplementary information

Supplementary Information

Description of additional Supplementary File

Supplementary Data 1

Supplementary Data 2

Reporting Summary

## Data Availability

A repository including data and scripts to replicate the analyses is available at this link: https://github.com/BIMIB-DISCo/oral_squamous_longitudinal.
