# Supplementary material for: Variant calling from scRNA-seq data allows the assessment of cellular identity in patient-derived cell lines
Source: Nat Commun. 2022 May 12;13:2718. doi: 10.1038/s41467-022-30230-w (PMC9098403; doi:10.1038/s41467-022-30230-w)
Supplement: Supplementary file 1 — Supplementary Information [file 41467_2022_30230_MOESM1_ESM.pdf]

Ramazzotti et al.
SUPPLEMENTARY INFORMATION: VARIANT CALLING FROM SCRNA-SEQ DATA ALLOWS THE ASSESSMENT OF CELLULAR IDENTITY IN PATIENT-DERIVED CELL LINES

ARISING FROM Sharma et al. *Nature Communications* `https://doi.org/10.1038/s41467-018-07261-3` (2018)

Additional materials and methods

GATK pipeline for variant calling from scRNA-seq data

To generate single-cell mutational profiles from scRNA-seq data, we employed the GATK Best Practices [2].

In particular, we selected the data uploaded on GEO repository with accession number `GSE117872`. As reported in the original work [6], single-cell RNA-seq data were generated via the C1 Single-Cell Auto Prep IFC (Fluidigm) system. First, cells were isolated within the 96 well plates. Then, cDNA syntesis and pre-amplification were performed using SMARTer and Advantage 2 kits (Clontech). Finally, Single-cell libraries were generated by using the Nextera DNA Sample Preparation Kit and the Nextera Index Kit (Illumina). Libraries univocally barcoded were pooled and sequenced with HiSeq-2500 sequencer (Illumina).

We downloaded the sample metadata by using the R library *GEOquery*. Single cells in the datasets were sequenced with two different library layouts and, in particular, all cell lines at each time point were sequenced using a *single-end* library layout, exception made for: (i) the HN137M (metastatic cell line before treatment), which includes 75 single cells sequenced with paired-end library layout; (ii) the HN137P (primary line before treatment), which includes 83 single cells sequenced with paired-end library layout and 170 with single-end library layout; moreover, 80 of such 170 cells were sequenced in two runs and are associated to two distinct FASTQ files.

We downloaded the RNA sequences using SRA toolkit. For each single cell sequenced in only one run with a single-end library layout, we obtained a single FASTQ file. For any single cell with double sequencing runs we concatenated the two FASTQ files. For cells with paired-end library layout, we only considered the forward reads files. As a result, we processed a single FASTQ file for each single cell of all datasets.

To summarize, the sample size for each dataset used in the analysis is the following: HN120P: 90 single cells, HN120PCR: 95, HN120PCRDH: 93, HN120M: 91, HN120MCR: 92, HN120MCRDH: 87, HN137P (*single-end*): 170 (80 of which with duplicated runs), HN137P (*paired-end*): 83, HN137PCR: 78, HN137PCRDH: 75, HN137M: 75, HN137MCR: 87.

We used Trimmomatic (v. 0.39) to remove the nucleotides with low quality score from the RNA sequences [1]. We then called SNVs and indels in single cells by employing the GATK Best Practices. In particular, we aligned the single cell reads on the human reference genome (GRCh38 release) using the STAR aligner in 2-pass mode [3]. Then, we used Picard tools to preprocess the SAM files by adding read groups, sorting, marking duplicates and indexing. We used GATK (v. 3.8.1) to hard clip intronic regions with SplitNCigarReads utility and to re-calibrate base alignment by using BaseRecalibrator utility. This step requires the information about known single nucleotide polymorphisms (SNPs), which we retrieved on the dbSNP 1000 genome project phase 3. Finally, we used HaplotypeCaller and VariantFiltration to call genotype variants and filter out those with low quality score (with default parameters).

After applying the GATK pipeline, we generated a VCF file for each single cell. We used Annovar [8] to annotate the variants (i.e., synonymous or non-synonymous SNV, frame-shift insertion or deletion, stop-gain or stop-loss) and to add

the rsID of each mutation, if available. Finally, we merged all the single-cell VCF files and applied custom filters as explained in the main text.

Single-cell transcriptomic analysis via Seurat

Single-cell transcriptomic analysis was performed via Seurat [7]. In particular, we processed the normalized data included in the original dataset (GEO online repository, accession code `GSE117872`). Data were log-scaled and Z-score normalized. The 1000 most variable genes were then selected by using the variance stabilizing transformation (VST) [4]. We finally computed the Principal Component Analysis on the selected genes and we employed the first 20 components to run the t-SNE algorithm for dimensionality reduction with default parameters [5].

References

[1] Anthony M Bolger, Marc Lohse, and Bjoern Usadel. Trimmomatic: a flexible trimmer for Illumina sequence data. *Bioinformatics*, 30(15):2114–2120, 2014.

[2] Mark A DePristo, Eric Banks, Ryan Poplin, Kiran V Garimella, Jared R Maguire, Christopher Hartl, Anthony A Philippakis, Guillermo Del Angel, Manuel A Rivas, Matt Hanna, et al. A framework for variation discovery and genotyping using next-generation DNA sequencing data. *Nature genetics*, 43(5):491, 2011.

[3] Alexander Dobin, Carrie A Davis, Felix Schlesinger, Jorg Drenkow, Chris Zaleski, Sonali Jha, Philippe Batut, Mark Chaisson, and Thomas R Gingeras. STAR: ultrafast universal RNA-seq aligner. *Bioinformatics*, 29(1):15–21, 2013.

[4] Blythe P Durbin, Johanna S Hardin, Douglas M Hawkins, and David M Rocke. A variance-stabilizing transformation for gene-expression microarray data. *Bioinformatics*, 18(suppl_1):S105–S110, 2002.

[5] Laurens van der Maaten and Geoffrey Hinton. Visualizing data using t-sne. *Journal of machine learning research*, 9(Nov):2579–2605, 2008.

[6] Ankur Sharma, Elaine Yiqun Cao, Vibhor Kumar, Xiaoqian Zhang, Hui Sun Leong, Angeline Mei Lin Wong, Neeraja Ramakrishnan, Muhammad Hakimullah, Hui Min Vivian Teo, Fui Teen Chong, et al. Longitudinal single-cell RNA sequencing of patient-derived primary cells reveals drug-induced infidelity in stem cell hierarchy. *Nature communications*, 9(1):4931, 2018.

[7] Tim Stuart, Andrew Butler, Paul Hoffman, Christoph Hafemeister, Efthymia Papalexi, William M Mauck III, Yuhan Hao, Marlon Stoeckius, Peter Smibert, and Rahul Satija. Comprehensive integration of single-cell data. *Cell*, 177(7):1888–1902, 2019.

[8] Kai Wang, Mingyao Li, and Hakon Hakonarson. Annovar: functional annotation of genetic variants from high-throughput sequencing data. *Nucleic acids research*, 38(16):e164–e164, 2010.
