# Supplementary material for: Variant calling from scRNA-seq data allows the assessment of cellular identity in patient-derived cell lines
Source: Nat Commun. 2022 May 12;13:2718. doi: 10.1038/s41467-022-30230-w (PMC9098403; doi:10.1038/s41467-022-30230-w)
Supplement: Supplementary file 2 — Description of additional Supplementary File [file 41467_2022_30230_MOESM2_ESM.pdf]

Descriptions of Additional Supplementary files

Supplementary Data 1 : Coverage and alternative allele read count are reported for each SNV and each cell included in the analysis.

Supplementary Data 2 : For each SNV included in the analysis and for each dataset, we report: genome position, reference and alternative alleles, rsID (if available), minor allele frequency (if available), the count and the ratio of single cells displaying the variant (coverage >= 5, alternative allele supporting read count >=3), the average coverage and the average number of supporting reads relative to the variant.
